# Remote training as a common tool for the different professionals involved in the acute phase after terror attacks across Europe: Perspectives from an expert panel

**DOI:** 10.3389/fpsyt.2022.915929

**Published:** 2022-08-23

**Authors:** Florence Askenazy, Arnaud Fernandez, Levent Altan, Michèle Battista, Michel Dückers, Morgane Gindt, Ophélie Nachon, Aleksandra Ivankovic, Ingeborg Porcar-Becker, Nathalie Prieto, Philippe Robert, Lise Eilin Stene, Susanne Thummler, Valeria Manera

**Affiliations:** ^1^Cognition Behaviour Technology (CoBTeK) Lab, Université Côte d’Azur, Nice, France; ^2^University Department of Child and Adolescent Psychiatry, Children’s Hospitals of Nice CHU-Lenval, Nice, France; ^3^Victim Support Europe, Brussels, Belgium; ^4^ARQ Centre of Expertise for the Impact of Disasters and Crises, Diemen, Netherlands; ^5^Nivel-Netherlands Institute for Health Services Research, Utrecht, Netherlands; ^6^Faculty of Behavioural and Social Sciences, University of Groningen, Groningen, Netherlands; ^7^Unit for Trauma, Crisis and Conflicts at the Autonomous University of Barcelona (UAB), Cerdanyola del Vallès, Spain; ^8^Cellule d’Urgence Médico-Psychologique, Centre Régional du Psychotraumatisme, Hôpital Edouard Herriot, Lyon, France; ^9^Association Innovation Alzheimer, Nice, France; ^10^Norwegian Centre for Violence and Traumatic Stress Studies (NKVTS), Oslo, Norway

**Keywords:** terror attack, acute response phase, remote training, information and communication technologies, SWOT analysis

## Abstract

The acute response after a terror attack may have a crucial impact on the physical and psychological wellbeing of the victims. Preparedness of the professionals involved in the acute response is a key element to ensure effective interventions, and can be improved through trainings. Today in Europe there is a recognized lack of inter-professional and international trainings, which are important, among others, to respond to the needs and the rights of victims affected by a terrorist attack in another country than their home country. In this paper we report the perspectives of an expert panel composed by different categories of professionals on the possible role of interprofessional trainings provided remotely. The experts discussed the pertinence of remote trainings for professionals involved in the acute response of a terror attack, and highlighted their Strengths, Weaknesses, Opportunities and Threats (SWOT analysis). We concluded that, while remote trainings cannot replace in-person trainings, they may be useful to share knowledge about the role and the organization of the different categories of professionals, thus potentially improving response coordination, and to easily share good practices across professionals and countries.

## Introduction

The acute response after a terror attack may have a crucial impact on the physical and psychological wellbeing of the direct victims (1–7). The acute phase management plays a key role also on the other impacted persons (family members or friends of the direct victims, members of their community, witnesses, members of the community where the attack took place, as well as a first responder and/or professional involved in the acute phase or in the follow-up (8–11). The acute phase can be defined as the phase during and immediately after the terror attack, and includes the initial steps of the rescue, identification, registration of all impacted persons, reunification, and immediate medical and psychological care (12–15). The duration of this phase depends on several factors (such as the type of event, the number of impacted persons, the rapidity of the response) and may vary from 1 week up to 1 month based on some definitions (16, 17). Several professionals are involved in the acute response phase, including first responders, such as law enforcement officers, paramedics, emergency medical technicians, firefighters, police, no-profit organization volunteers; and different categories of clinicians, such as medical doctors, psychologists, psychotherapists, and nurses (12, 18, 19). All these different professionals need to accomplish their respective tasks under uncertain, often chaotic conditions, and need to coordinate with other professionals, meaning that, at minimum, they should know their respective roles (National Collaborating Centre for Mental Health (11, 19–21)). Furthermore, persons impacted from terror attacks can be foreign tourists or visitors, meaning that they do not necessarily speak the country language and/or have the same social support, health coverage, or juridical and compensation systems. In this context, the European Commission financed the creation of a EU Centre of Expertise for Victims of Terrorism (EUCVT), which aims to bring together different categories of international experts of terrorism and provide European guidelines and trainings to increase knowledge on the victim needs and rights.

Preparedness of the professionals to face mass trauma is a key element, as recognized by professionals themselves (22, 23). Professional training is recognized as the best way to help them to prepare for the emergency response, including the implementation of frequent and realistic planning and training drills (24). Furthermore, several guidelines for psychosocial support stress the importance of training to contribute to preparedness (see (25) for a recent review). However, there is a recognized lack of inter-professional trainings, as well as a lack of international trainings. This is especially important because there is a variability in the level of preparedness in dealing with terrorism across countries (6, 15, 26, 27). Analyses of disaster preparedness definitions typically point at a lack of consensus (28, 29). Definitions depend on the purpose of a study. In our case, we focus specifically on disaster preparedness during the response phase of a threatening situation. We interpret preparedness as a state of professional readiness in the response, reflected in the ability to localize affected people, awareness of their potential and actual immediate support needs and risks, knowledge on how to provide basic support, on the availability of services and how to access them, through traditional and innovative methods, in the context of the support system.

The advances of new Information and Communication Technologies (ICT) allows today to organize trainings remotely, which can be more time and cost effective. Remote professional trainings are more and more widely used also outside universities and classical teaching facilities, especially after the COVID-19 sanitary crisis, and are usually well-accepted (30, 31). They can contribute, among others, to continuing education. Some disadvantages can also be highlighted compared to in-person trainings, including the difficulty to perform role-playing exercises, the reduced interpersonal contact, and lower engagement.

Most of the published evidence collected on training efficacy in the domain of response preparedness focuses on face-to-face trainings (e.g., (32–34)). However, online trainings do also exist, for instance for the continuing education of the law enforcement officers (35, 36). Accelerated by the COVID-19 crisis, online trainings are becoming more common also in the domain of disaster preparedness (e.g., (37)). As online trainings are gaining popularity only recently in this domain, analyses of their advantages and disadvantages are needed. In the present paper, we describe the perspectives discussed by a panel of experts in the context of the EUCVT project concerning the topics that should be taught during inter-professional and international trainings, and the role of remote trainings for the professionals involved in the acute response phase of a terror attack.

## Methods

The discussion panel was initiated by the Lenval Foundation and the CoBTeK lab of the Université Cote d’Azur. Based on the collaborative network established in previous projects in the domain of psychotrauma due to terror attacks (such as the EUCVT project), we initially contacted a list of twenty-one internationally recognized experts (hereafter called “core experts”) in the field of psychosocial support in the acute response phase from different countries. These included (more than one response was possible) researchers (*N* = 14), clinicians (e.g., psychiatrists, psychologists, *N* = 17) and members of victim support organizations and/or organizations working on psychotrauma/acute response coordination (*N* = 12). These experts worked in Belgium (*N* = 2), France (*N* = 11), Israel (*N* = 1), Netherlands (*N* = 2), Norway (*N* = 1), Spain (*N* = 1), the United Kingdom (*N* = 2) and United States (*N* = 1). They had at least 15 years of professional experience in works related to psychotrauma and traumatic stress (range: 15 years to more than 30 years). All of them had direct experience with the acute response after terrorist attacks.

These core experts helped to define the initial topics and questions, to decide the questions for the web-survey, and/or provided short presentations concerning the organization of the first response protocols in different countries, and/or moderated group sessions during a final plenary meeting. The 11 experts that initiated the expert panel (and were thus more directly involved in the survey creation), were not asked to respond to the questions. To obtain responses from a wider and diverse range of experts at the international level, the core experts shared the survey some professional networks, including the online Hub of Expertise launched by the EU Centre of Expertise for Victims of Terrorism (EUCVT). The extended group of experts (including 10 of the initial core experts that were not directly involved in the survey design, and 26 experts reached through EUCVT hub, for a total of 36 responders) were asked to answer questions via a web-survey (between April and June 2021) using Google Forms. After collecting the results, a facilitator (VM) provided a summary of the experts’ responses, and encouraged the experts to analyze, comment and (eventually) revise their earlier responses considering the commentaries of other members of the panel.

The survey included a section with information on the professional profile and experience of the responders (as detailed in the Participants section), followed by rating questions focused on the design of international and interprofessional trainings (see Table 1). Rating questions employed a 7-point Likert scale (1 = Strongly disagree; 2 = Moderately disagree; 3 = Slightly Disagree; 4 = Neutral; 5 = Slightly agree; 6 = Moderately agree; 7 = Strongly agree). Mean and standard deviation were employed for the data analysis. The listed topics were derived from those described in European disaster mental health guidelines and evaluation studies, including registration of information on affected people, screening/detection of individuals in need for support or at risk, use of basic psychosocial support tools, availability of services, referral to appropriate support providers, how to deal with particular vulnerable or risk groups (e.g., children, people with disabilities), characteristics of the support system for people affected and mental health risks for professionals themselves (11, 12, 21, 38, 39). Topics like these can be considered logical candidates to include in trainings for professionals. Moreover, since new interventions and healthcare technologies are being developed and tested, including ICT, such as online platforms and Virtual Reality, it makes sense to include a rating question on their relevance for an emergency response training for professionals. After each rating question, participants could provide open comments. Finally, the survey included open questions, in which the experts were asked to provide a list of the top 3 advantages and disadvantages of using ICT in the acute response phase and of using ICT for the training of the professionals involved in the acute response phase. These were used to make a SWOT analysis (Strengths, Weaknesses, Opportunities and Threats) of the use remote trainings for professionals involved in the acute response phase of a terror attack, as reported in Table 2. The web-survey results and the open discussion points were revised by the core experts (*N* = 21) and the extended list of experts (*N* = 26) during a hybrid plenary meeting held on July 16th, 2021, in Nice (France). 11 out of the 47 experts were physically present in Nice, while the others were connected remotely.

**TABLE 1:** Results of the rating questions.

It is necessary that all the professionals involved in the acute phase (including first responders) know	*N* = 36 Mean (SD)
How/where to register the information of the impacted people for future tracking	6.8 (0.5)
How to screen/detect individuals that need specific psychosocial support	6.3 (0.9)
How to use basic tools for psychosocial support (such as relaxation, etc.)	5.6 (1.7)
All the services available for victims (social, legal, medical, etc.)	6.0 (1.2)
Where and toward whom referring victims who need psychosocial support	6.6 (0.7)
How to identify, handle and/or orient vulnerable victims (children, people with disability, foreigners, pregnant women, etc.)	6.5 (0.9)
That each European country has its own system to support their citizens	5.5 (1.4)
That they are themself at risk for PTSD and other psychiatric disorders	6.6 (0.7)
ICT should be used to train the professionals involved in the emergency response phase (e.g., online platforms, Virtual Reality)	5.7 (1.5)

Rating questions employed a 7-point Likert scale (1 = Strongly disagree; 2 = Moderately disagree; 3 = Slightly Disagree; 4 = Neutral; 5 = Slightly agree; 6 = Moderately agree; 7 = Strongly agree).

**TABLE 2:** Training of professionals in the acute phase: SWOT Analysis of using ICT.

Strengths	Weaknesses
• Allow to train large numbers of persons • Cost and time effective • Can be used everywhere • Training standardization (objectives, methods, trainer) • Can be recorded, and repeated • Can be easily updated • Can be translated in different languages • Possibility to have training modules specific to certain professionals • Easy adaptation to the pre-training level (proposition of different modules) • Easy to verify which information the learner acquired • Can improve exchanges, coordination, and harmonization between different categories of professionals • Can improve international cooperation, coordination, and harmonization • Possibility to train in realistic settings, and possibility to use simulation (e.g., VR) • Possibility to create interactive trainings (e.g., interactive videos) which improve learning and increase motivation	• Need of devices/equipment (logistics) • Need of maintenance and update • May be difficult for older professionals • Need of trainings to learn how to use E-learning devices and platforms • For recorded trainings, lack of social interaction, customization, and personalized feedback • Difficult to create a group dynamic • Difficult to train social cohesion, interpersonal skills, and inter-professional support • May diminish concentration and motivation • Less adapted for “real-life” group trainings and simulations • Risk of teaching standardized, inflexible strategies and responses (less experience working with uncertainty) • Difficult to be emotionally involved, and thus learn how to regulate emotions and stay rational in stressful situations • Reduced vicarious learning

**Opportunities**	**Threats**

• Rapid development on E-learning platforms • Competition between E-learning platforms should make them cheaper, more reliable, and user-friendlier • Technology (e.g., VR) becoming cheaper and more widespread • Increase in the number of people worldwide that have access to the internet • Acceleration in the acceptability of E-learning (due to COVID-19 pandemics) • Increase in the number of people that follow online trainings (in all professional categories) • Recommendations emerging on how to design motivating online trainings	• Initial financial cost for institutions • Technology not always reliable (e.g., internet coverage, equipment failure) • Lack of familiarity with new technologies for teachers and learners • First responders are reluctant in advising to switch to online learning, potentially limiting its impact/use • Risk of disparities between countries and/or regions • Reduction of the number of real-life simulations in favor of online trainings • Teachers may be reluctant to record/share online trainings

### Participants

The survey was completed by 36 international experts working on trauma/traumatic stress. 94% of them (*N* = 34) reported to have direct experience with the acute response after terrorist attacks in the following countries: Belgium, France, Germany, Finland, Israel, Luxemburg, Norway, Portugal, Spain, United Kingdom. The years of professional experience ranged from 2 to 34 (mean = 16 years, SD = 9 years). The experts included (more than one response was possible): clinicians (89%; *N* = 32), including psychologists, psychiatrists, and medical doctors; researchers (42%; *N* = 15) in the domains of psychology, psychiatry, child and adolescent psychiatry, forensic science, public health, sociology/suicidology, or education; first responders (25%; *N* = 9), including emergency medical technicians, psychologists, psychiatrists, NGO representatives, psychosocial emergency services, policemen and nurses; and people working in victim support organizations (25%; *N* = 9). In addition to terror attack, the experts worked with the following types of traumas: Single trauma (89%, *N* = 32), Disaster (89%, *N* = 32), Death/Bereavement (83%, *N* = 30), Repeated Trauma (81%, *N* = 29), Child Abuse/Maltreatment (75%, *N* = 27), Vicarious Traumatization in Professionals/Helpers (67%, *N* = 24), Rape/Sexual Assault (64%, *N* = 23), Refugee/Displacement Experiences (56%, N = 20), Intimate Partner Violence (53%, *N* = 19), War/Post-Conflict Settings – Civilians (50%, *N* = 18), Torture (50%, *N* = 18), Community Violence (47%, *N* = 17), Medical Trauma (47%, *N* = 17), Racism/Historical Trauma (25%, *N* = 9), War – Military/Peacekeepers/Veterans (36%, *N* = 13). In terms of populations, the experts had professional experience working with adults (89%, *N* = 32), adolescents (78%, *N* = 28), children (67%, *N* = 24), seniors (39%, *N* = 14), people with special needs (25%, *N* = 9), and babies/toddlers (22%, *N* = 8), Overall, 67% of the experts (*N* = 24) declared to be involved in the organization of specific trainings on acute post-disaster psychosocial care in their country.

## Results

The results of the rating questions concerning the topics that are important to include into inter-professional and international trainings are reported in Table 1. As a group, the 36 experts that responded to the survey strongly agreed (mean score greater than 6) that it is necessary that all the professionals involved in the acute phase know how/where to register the information of the impacted people for future tracking, how to screen/detect individuals that need specific psychosocial support, where and towards whom referring victims who need psychosocial support, how to identify, handle and/or orient vulnerable victims (for instance children, people with disability, foreigners, pregnant women), and that they are themself particularly at risk for PTSD and other psychiatric disorders. The experts moderately agreed (scores between 5 and 6) that all the professionals should know how to use basic tools for psychosocial support (such as relaxation), that they should be aware of all the services available for victims (e.g., social, legal, medical), and that each European country has its own system to support their citizens. Furthermore, they moderately agreed on the fact that new ICT, such as online platforms and Virtual Reality, should be used to train the professionals involved in the emergency response phase. In the open comments, the experts stressed that, despite most of these topics are included in existing trainings, it is important that all professionals have common knowledge, to facilitate inter-professional and international exchange. These general trainings are meant to complement – and not to replace - the trainings organized at the national and local level, and those organized for specific categories of professionals, which provide important action plans that are location- and profession- specific.

Concerning the analysis of the positive and negative aspects of organizing remote trainings for professionals involved in the acute response phase of a terror attack, we performed a SWOT analysis based on the open comments provided by the 36 experts that responded to the survey, and by the comments provided during the plenary meeting by all the 47 experts. The results are reported in Table 2.

### Strengths

The experts recognized that, as to all professional trainings, remote trainings for professionals involved in the acute response phase can allow to train large numbers of persons everywhere, being thus cost-and time-effective. They suggested that trainings can be recorded, repeated, updated, and translated in different languages (or subtitled), thus allowing training standardization in terms of objectives, methods, and trainers. Similarly, remote trainings offer the possibility to design training modules specific to certain categories of professionals and customized to the pre-training level. In the experts’ opinion, remote trainings can improve exchanges, coordination, and harmonization between different categories of professionals and can improve international cooperation, coordination, and harmonization (or, at minimum, increase the awareness that different countries have in place different regulations and procedures). Furthermore, the experts highlighted that it may be quite easy to verify (e.g., via a multiple-choice questions) which information the learner acquired, at least in terms of explicit knowledge. They advanced that it is possible, even if it requires specific competence, to design trainings in more realistic settings, for instance using Virtual Reality or Augmented Reality applications, and to create interactive trainings (e.g., interactive videos) which may improve learning and increase motivation.

### Weaknesses

The experts acknowledged that remote trainings for professionals involved in the acute response phase have the same limitations as those for other professionals in terms of technical requirements, but have also some specificities. As any other remote training, there is a need of devices/equipment (logistics), of maintenance and update, and of learning how to use E-learning devices and platforms. This is especially true for professionals not familiar with new technologies, such as elderly people. The experts suggested that, especially for recorded offline trainings, there is a lack of social interaction, customization, and of personalized feedback, which may diminish concentration and motivation to learn. Vicarious learning may also be also reduced. The experts acknowledged that group dynamics may be more difficult to create. This is particularly important for professionals involved in the acute response phase because they must work on the sense of social cohesion, on interpersonal skills, and inter-professional support. They also highlighted a potential risk of teaching standardized, inflexible strategies and responses, thus reducing the self-awareness of what happens when working under uncertainty and complex, evolving situations. Indeed, in remote trainings it may be harder to become emotionally involved due to the physical distance among participants, and thus participants may have a reduced experience in learning how to regulate emotions and stay rational in stressful situations. In the experts’ opinion, the ability to work in chaotic, stressful settings can be better trained using role-playing and simulation training methods.

### Opportunities

The experts acknowledged that, due to the COVID-19 crisis, we are experiencing an acceleration of the development on E-learning platforms from different producers, resulting in cheaper, more reliable, and user-friendlier interfaces. Similarly, the acceptability on online trainings has increased, as both teachers and learners understood the advantages of remote learning (time and cost efficiency, possibility to customize, etc). In parallel, there is an increase worldwide in the number of people that have access to the internet, and technologies are becoming cheaper and more widespread, including VR and other applications to make trainings more immersive and interactive. Importantly, the experts recognized that recommendations are now emerging on how to design motivating online trainings, which are promising for the future of remote teaching (40).

### Threats

The experts highlighted that creating high-quality remote trainings may have an initial financial cost for institutions and may be time-consuming, thus limiting the number of trainings that are developed. This may be worsened by the lack of familiarity with new technologies for teachers and learners. In addition, the experts recognized that technologies are not always reliable (e.g., internet coverage, equipment failure), limiting actual use of remote trainings. Based on the expert panel expertise, first responders may be reluctant in advising to switch to online learning, potentially limiting its impact/use. Other listed threats included the risk of disparities between countries and/or regions depending on the internet coverage, and the fact that teachers may be reluctant to record/share online trainings, especially professional teachers that teach as a job. In addition, the reduction of the number of real-life simulations in favor of online trainings may be risky, as role-playing and simulations are considered as essential part of the training for the professionals involved in an emergency response.

## Discussion

The expert panel in the context of EUCVT network, including researchers and different categories of professionals working with victims of terror attacks, agreed on the importance of designing international and inter-professional trainings. These training should at least include information on the initial triage, registration, screening, first support, and referral of victims to the right professionals and services, with a special attention to vulnerable victims and victims that were affected by a terrorist attack in another country than their home country. In addition, interprofessional trainings should include modules warning on the increased risk of developing PTSD and other psychiatric disorders for the professionals involved in the acute response phase, and on how to detect potential symptoms and apply selfcare. Despite these topics are included in trainings, the experts acknowledged that it is crucial to get a common ground among professional and countries, with the objective to complement existing national, local and professional-specific trainings, and facilitate the inter-professional exchange. Concerning the use of remote training platforms, converging with previous guidelines (e.g., (41)), the experts highlighted several advantages and opportunities, including the possibility to train more professionals everywhere, possibly sharing the same information across different professionals and countries. This is harder to do with classical training methods, due to logistic complexity. Despite the clear interest, remote trainings have several limitations, and should not replace completely physical trainings. The experts highlighted that real event simulations, role-playing and group exercises are crucial for professionals involved in terror attacks, and should not be replaced, and used in complement with new technologies such as Virtual Reality and Augmented Reality that, when confirmed to be reliable and effective, will be made available to the wide public. Hybrid training formats, combing online trainings for acquiring more theoretical knowledge (know what) and in person trainings to get first-hand experience on emotion regulation, decision making in stressful situation, and interpersonal cooperation (know how) should be favored. While these considerations are valid for all professional trainings, it is important to highlight the strengths and weaknesses of online trainings in the domain of preparedness for the emergency response phase to allow this specific field to grow more rapidly, and design trainings involving different professionals and countries. It would be important to verify if the perspective provided in this paper are shared by larger groups of professionals, as well as to compare the perspectives of different professionals and, even more, to broaden the scope up to victims of other potentially traumatic events and people exposed to disasters. Finally, any form of training can only fulfill its promise of a strengthened service delivery when it is implemented effectively. Among the implementation factors to consider, it is highly likely that the culture and supportive setting within organizations are linked to the motivation, capability, and opportunity of trainees to effectively participate in remote learning and the practical application of the training content.

## Data availability statement

The original contributions presented in this study are included in the article/supplementary material, further inquiries can be directed to the corresponding author.

## Author contributions

FA and VM coordinated the expert panel. VM, FA, ON, MG, and ST wrote the first draft of the manuscript. All authors of the manuscript participated to the expert panel organization and discussions, and contributed to the manuscript drafting and revision.
